# Chromosome microduplication in somatic cells decreases the genetic stability of human reprogrammed somatic cells and results in pluripotent stem cells

**DOI:** 10.1038/srep10114

**Published:** 2015-05-12

**Authors:** Yang Yu, Liang Chang, Hongcui Zhao, Rong Li, Yong Fan, Jie Qiao

**Affiliations:** 1 Center of Reproductive Medicine, Department of Obstetrics and Gynecology, Peking University Third Hospital, Beijing, 100191, China; 2 Key Laboratory of Assisted Reproduction, Ministry of Education, Beijing, 100191, China; 3 Beijing Key Laboratory of Reproductive Endocrinology and Assisted Reproductive Technology, Beijing, 100191, China; 4 Key Laboratory for Major Obstetric Diseases of Guangdong Province, the Third Affiliated Hospital of Guangzhou Medical University, Guangzhou, 510150, China

## Abstract

Human pluripotent stem cells, including cloned embryonic and induced pluripotent stem cells, offer a limitless cellular source for regenerative medicine. However, their derivation efficiency is limited, and a large proportion of cells are arrested during reprogramming. In the current study, we explored chromosome microdeletion/duplication in arrested and established reprogrammed cells. Our results show that aneuploidy induced by somatic cell nuclear transfer technology is a key factor in the developmental failure of cloned human embryos and primary colonies from implanted cloned blastocysts and that expression patterns of apoptosis-related genes are dynamically altered. Overall, ~20%–53% of arrested primary colonies in induced plurpotent stem cells displayed aneuploidy, and upregulation of P53 and Bax occurred in all arrested primary colonies. Interestingly, when somatic cells with pre-existing chromosomal mutations were used as donor cells, no cloned blastocysts were obtained, and additional chromosomal mutations were detected in the resulting iPS cells following long-term culture, which was not observed in the two iPS cell lines with normal karyotypes. In conclusion, aneuploidy induced by the reprogramming process restricts the derivation of pluripotent stem cells, and, more importantly, pre-existing chromosomal mutations enhance the risk of genome instability, which limits the clinical utility of these cells.

Pluripotent stem cells have tremendous potential in regenerative medicine and cell replacement therapy based on their self-renewal and multi-differentiation characteristics under specific conditions^1^. To overcome the immunological rejection that often occurs when exogenous cells or tissues are transplanted into the host, two methods have been developed: somatic cell nuclear transfer (SCNT) technology to produce nuclear transfer embryonic stem cells (NT-ES cells) and forced ectopic expression of defined transcription factors in somatic cells to produce induced pluripotent stem cells (iPS cells). Pluripotent stem cells have been successfully derived in multiple species, including mouse, monkey and human, and they represent potential resources for cell therapy. However, their low efficiency of derivation generally limits their further application in the clinic.

NT-ES cells were first successfully established in mouse in 2001^2^. Although lower full-term development efficiency was reported in cloned mice, NT-ES derivation efficiency was similar to that of normal ES cells from fertilized blastocysts, indicating development potential comparable to that of the inner cell mass (ICM) of cloned blastocysts. The first NT-ES cell line was derived from a rhesus monkey, a non-human primate, in 2007^3^. The study showed only 6% derivation efficiency from cloned monkey blastocysts, which was significantly lower than that from normal fertilized embryos. The researchers suggested that epigenetic modification during somatic cell reprogramming by oocytes contributed to the lower efficiency (with an almost three-fold difference in NT-ES derivation) in monkeys^4^. In 2013, human NT-ES cells were successfully obtained, considered a significant milestone in therapeutic cloning^5^. Notably, the protein phosphatase inhibitor caffeine appears to be necessary for NT-ES derivation. Although a higher success rate for NT-ES derivation has been reported in that study, actual efficiency is still low if the rate is calculated based on the number of oocytes rather than blastocysts, indicating that key factors at early stages in the development of cloned embryos affect NT-ES derivation.

Yamanaka and co-workers initially reported the successful application of iPS cell technology in mouse^6^, and subsequently in rat^7^, monkey^8^ and human^9^. At the initial stage, efficiency was extremely low, and only one iPS cell could be collected from 1,000–10,000 cells. Following the use of microRNA to induce the conversion of somatic cells into iPS cells, efficiency was increased 100-fold^10^. Small compounds and drug-like molecules were also utilized for iPS cell production, with consequent enhancement of derivation efficiency^11^,^12^. Overexpression of Mbd3, a subunit of NuRD, inhibited induction of iPSCs. Conversely, depletion of Mbd3 improved reprogramming efficiency, resulting in deterministic and synchronized iPS cell reprogramming (nearly 100% efficiency within 7 days from mouse and human cells)^13^,^14^.

Chromosome division error in cell mitosis results in “daughter” cells having the incorrect number of chromosomes. An extra or missing chromosome contributes to developmental failure or disease in offspring. Even micro-deletion or micro-duplication is suggested to play an important role in human development. Muune *et al.* indicated that only 13% lower-quality embryos show diploid chromosomes^15^. In a study of SCNT, Yu *et al.* showed that micronuclei in cloned embryos are induced when the microinjection method is used instead of electrofusion, suggesting increased risk of chromosomal aberration by nuclear transfer technology^16^. Rapid propagation may induce karyotypic abnormalities in cultures of either embryonic stem cells (ES cells) or iPS cells. Taapken *et al.* showed the appearance of small chromosome segments during somatic reprogramming using iPS technology, indicating that chromosome aberrations are induced not only by SCNT but also by iPS technology^17^. Although chromosomal mutations are suggested to occur during the reprogramming process, it is unclear whether they are associated with the low efficiency of somatic cell conversion into pluripotent stem cells.

In the present study, we investigated the effects of reprogramming technology via SCNT and iPS cells on the chromosomal stability of somatic cells, assessed the incidence of aberrant chromosomes using SNP screening methods, and discuss the association between chromosomal mutation and stem cell formation.

## Results

### Somatic cell chromosome karyotyping and SNP analysis

**Figure 1:**
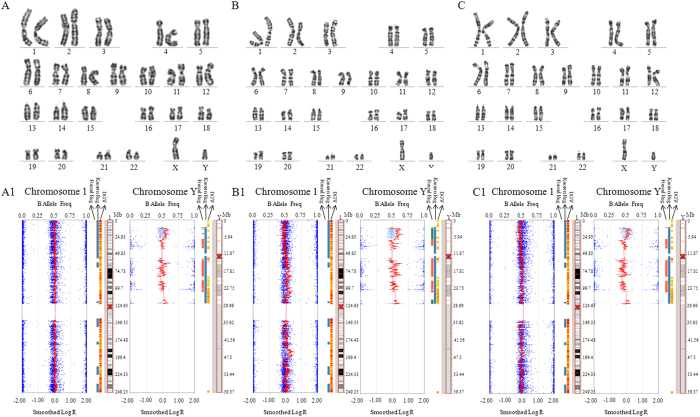
Chromosome identification for the serum samples of somatic cell donors. (**A**) Donor 1 with the cytogenetic method; (**A1**) Donor 1 with the SNP method; (**B**) Chromosomes 1 and Y in Donor 2 with the cytogenetic method; (**B1**) Chromosomes 1 and Y in Donor 2 with the SNP method; (**C**) Donor 3 with the cytogenetic method; (**C1**) Chromosomes 1 and Y in Donor 3 with the SNP method.

**Figure 2:**
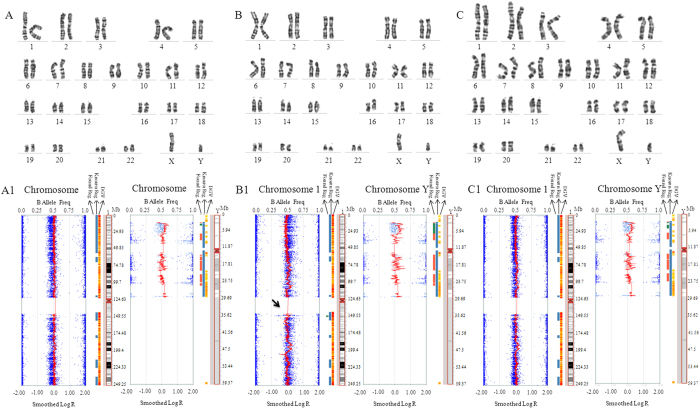
Chromosome identification for somatic cells from the biopsied skin of three donors. (**A**) Somatic cell 1 (**S1**) with the cytogenetic method; (**A1**) Chromosomes 1 and Y in S1 with the SNP method; (**B**) Somatic cell 2 (**S2**) with the cytogenetic method; (**B1**) Chromosomes 1 (dup(1)(q21.1q21.2)) (shown by black arrow) and Y in S2 with the SNP method; (**C**) Somatic cell 3 (**S3**) with cytogenetic method; (**C1**) Chromosomes 1 and Y in S3 with the SNP method.

Cellular karyotyping and SNP analysis of blood indicated no chromosomal mutations in all donors (Fig. 1 and Table S1). Somatic cells (S-1, S-2 and S-3) from three donors were used. All somatic cells showed normal cellular karyotypes at passage 10 (Fig. 2A–C). SNP results revealed a 2.4 Mb duplication in chromosome 1 in the S-2 cells (Fig. 2A1–C1 and Table S1) at passage 10, however no mutation was found at passage 3 (Table S1). Total 24 genes were located in this section (Fig. S1), and listed in Table S2. The microduplication in S-2 cells was reconfirmed using next-generation sequencing (NGS) methods (Table S3).

### SCNT embryos and primary colony derivation

**Figure 3:**
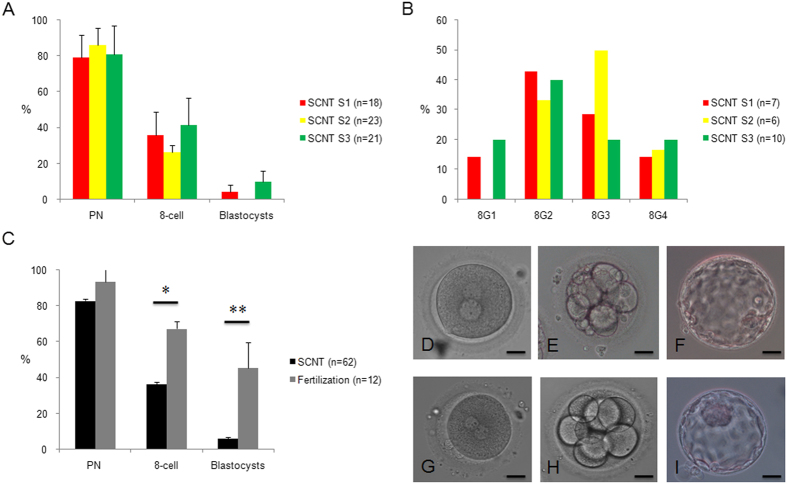
Summary of the development and quality of cloned and fertilized embryos. (**A**) Similar development efficiency of cloned embryos from different somatic cells in SCNT; (**B**) No significant differences in eight-cell grading were observed, although the number of high-grade embryos was lower in the SCNT-S2 group; (**C**) Lower efficiency of eight-cell and blastocyst formation was observed for cloned embryos than for fertilized embryos; * *P *< 0.05, and ** *P*  < 0.01; (**D**) Pseudo-pronuclei in cloned embryos after artificial activation; (**E**) Cloned embryo at the eight-cell stage; (**F**) Cloned embryo at the blastocyst stage; (**G**) Pronuclei in fertilized embryos; (**H**) Fertilized embryo at the eight-cell stage; (**I**) Fertilized embryo at the blastocyst stage. Bar represents 50 μm.

Using S-1, S-2 and S-3 cells, human cloned embryos were reconstructed. In total, 65 human oocytes were obtained and enucleated using Oosight software. After SCNT, 62 human oocytes survived, among which 52 were activated successfully, displaying one or two pseudo-pronuclei. Overall, 23 cloned embryos developed to the eight-cell stage from which four blastocysts were derived. No significant differences in development competence and eight-cell embryo grading were evident among cloned embryos from different donor somatic cells, although S-2 cells showed slightly worse outcomes (Fig. 3A,B). One and three blastocysts were obtained from S-1 and S-3 cells, respectively, but none were obtained from S-2 cells (Fig. 3A). ICSI manipulation of 12 human oocytes was performed as a control; eight of these embryos developed to the eight-cell stage, and five blastocysts were derived from 11 zygotes. The efficiencies of eight-cell and blastocyst formation were significantly higher than those of the corresponding stages in cloned embryos, although we observed no marked differences at the pronuclear stage (Fig. 3C). All eight-cell embryos from ICSI zygotes were graded as 8G1 (3) or 8G2 (5). Representative images showing the development of cloned and fertilized embryos are shown in Fig. 3D–I.

**Figure 4:**
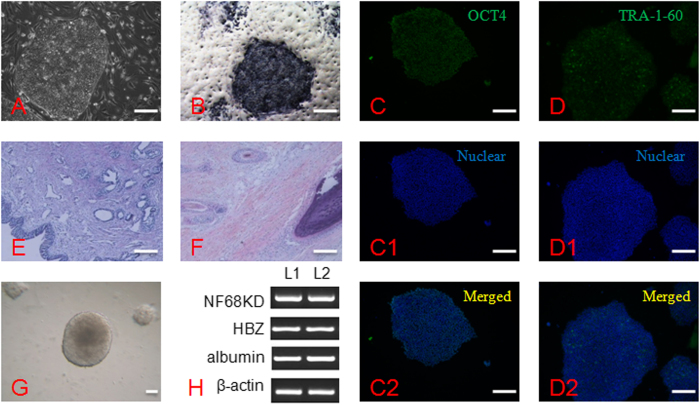
Characteristics of human embryonic stem cells from fertilized blastocysts. (**A**) ES cell colony on feeder cells; (**B**) AP activity (Black colony); (**C**-**C2**) OCT4 and nuclear staining, and merged images; (**D**-**D2**) TRA-1-60 and nuclear staining, and merged images; (**E**) Glandular epithelial cell (endoderm) from *in vivo* differentiated teratoma; (**F**) Cartilage (mesoderm) and hair follicles (ectoderm) from *in vivo* differentiated teratoma; (**G**) Embryoid body (EB) formation differentiated from ES cells *in vitro*; (**F**) Markers in all three germ layers were expressed in EBs, including NF68KD (ectoderm), HBZ (mesoderm) and albumin (endoderm). L1, human fertilized ES line 1; L2, human fertilized ES line 2.

Subsets of TE cells of blastocysts were biopsied and lysed for CGH testing, and the ICM was placed on feeder cells. Although primary colonies initially displayed normal growth, growth of the four cloned blastocysts was arrested after 5 days. Two ES cell lines were derived from five fertilized blastocysts. ES cells displayed normal morphology and AP activity, and they positively expressed a stem cell surface marker (TRA-1-60) and a pluripotent cell marker (OCT-4). Furthermore, the stem cells differentiated into derivatives from the three germ layers both *in vitro* and *in vivo* (Fig. 4).

### Embryonic chromosome identification and correlation analysis

**Figure 5:**
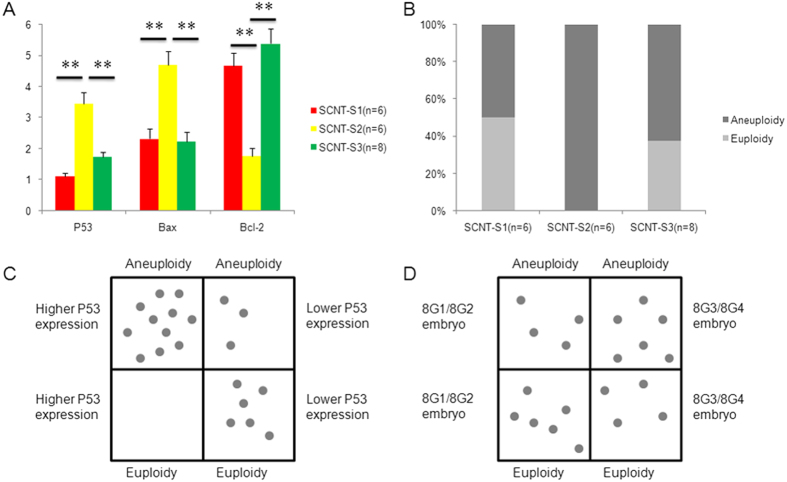
Apoptosis-related gene expression and correlation with chromosome euploidy in arrested cloned eight-cell embryos from different somatic cells. (**A**) Expression profiling of P53, Bax and Bcl-2 genes; ** *P* < 0.01; (**B**) Half of the embryos (50%) in the SCNT-S1 group, 100% of embryos in the SCNT-S2 group, and 60% of embryos in the SCNT-S3 group showed aneuploidy; (**C**) P53 gene expression levels were positively correlated with chromosome aneuploidy in cloned embryos; (**D**) Morphology was not correlated with chromosome aneuploidy in cloned embryos.

All embryos arrested at the eight-cell stage in each SCNT group were collected and biopsied (six embryos in SCNT-S1, six in SCNT-S2 and eight in SCNT-S3). For each embryo, half the blastomeres were examined for gene expression and half were examined for chromosome euploidy. Expression profiling of induced apoptotic genes revealed upregulation of P53 and Bax and downregulation of the anti-apoptotic gene Bcl-2 (Fig. 5A). In SNP analysis, cloned embryos showed varying degrees of aneuploidy in all 23 chromosomes (Fig. 5B). Moreover, correlations were observed among patterns of eight-cell morphology, gene expression and aneuploidy. Higher P53 gene expression, but not morphology, was positively correlated to embryo aneuploidy (Fig. 5C,D). Interestingly, chromosome segment duplication in somatic cells was not altered upon reprogramming via SCNT, and all embryos from S-2 cells displayed at least the 2.4 Mb duplication in chromosome 1. In the control group, only one of the three arrested eight-cell embryos showed slight chromosome duplication and deletion.

**Figure 6:**
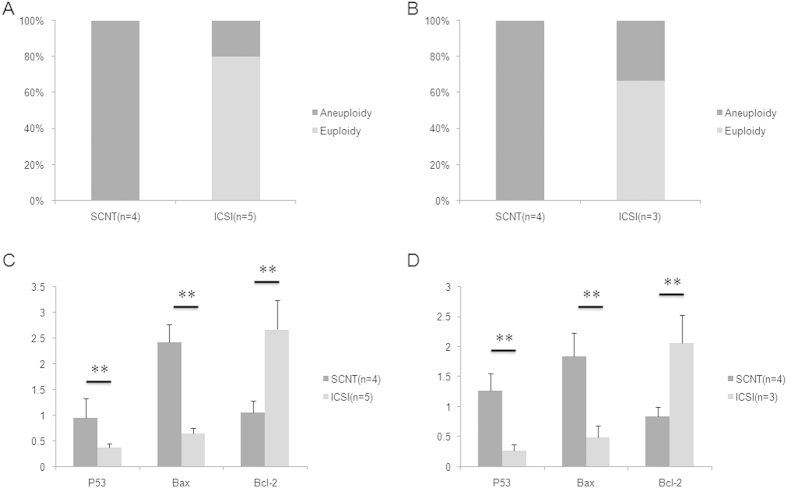
Expression of apoptosis-related genes and chromosome euploidy in trophectoderm (TE) cells and arrested primary colonies from cloned blastocysts. (**A**) All TE cells from cloned blastocysts displayed microdeletion/duplication in certain chromosomes, whereas 80% cells from ICSI blastocysts had normal euploid chromosomes; (**B**) TE cells from cloned blastocysts showed significant increases in P53 and Bax expression and significant decreases in Bcl-2 expression; ** *P*  < 0.01; (**C**) All arrested primary colonies from cloned blastocysts showed microdeletion/duplication in certain chromosomes, but the aneuploidy ratio in the arrested primary colonies from ICSI blastocysts was 33.3%; (**D**) Arrested primary colonies from cloned blastocysts showed significant increases in P53 and Bax expression and significant decreases in Bcl-2 expression; ** *P*  < 0.01.

All TE cells from cloned blastocysts displayed aberrant chromosome segments, although the aberrant region was less than 5 Mb, and it was screened using SNP array only. However, only one of the fertilized blastocysts showed this chromosomal abnormality, whereas the others were normal (Fig. 6A). RT-PCR results similarly indicated higher P53 and Bax and lower Bcl-2 mRNA levels in TE cells of cloned blastocysts than in those of fertilized blastocysts (Fig. 6B). We further examined aneuploidy and aberrant gene expression in arrested primary colonies from both cloned and fertilized blastocysts. Similar results were obtained, with higher aneuploidy rates and expression P53 and Bax genes expression in cloned primary colonies than in fertilized primary colonies (Fig. 6C,D).

### iPS cell derivation and identification

**Figure 7:**
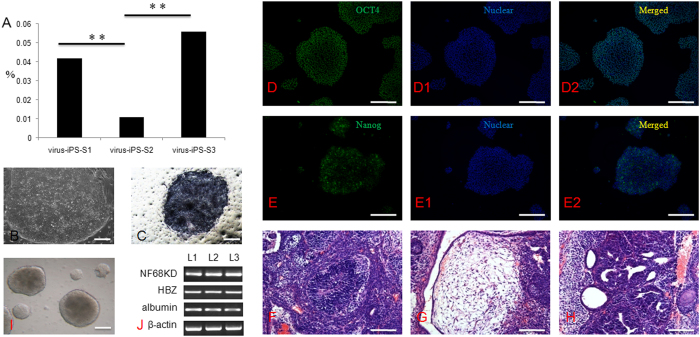
Derivation and characteristics of iPS cells from different somatic cells. (**A**) Derivation efficiency was significantly decreased when S2 somatic cells were transfected with virus; (**B**) iPS cell colony on feeder cells; (**C**) AP activity (Black colony); (**D**–**D2**) OCT4 and nuclear staining and merged images; (**E**–**E2**) TRA-1-60 and nuclear staining and merged images; (**F**) Neural rosette (ectoderm) from *in vivo* differentiated teratoma; (**G**) Fat tissue (Mesoderm); (**H**) Primitive gut (endoderm) from *in vivo* differentiated teratoma; (**H**) Embryoid body (EB) formation differentiated from ES cells *in vitro*; (**I**) Markers in all three germ layers were expressed in EB, including NF68KD (ectoderm), HBZ (mesoderm) and albumin (endoderm). L1, human iPS cells from S1 somatic cells; L2, human iPS cells from S2 somatic cells; L3, human iPS cells from S3 somatic cells.

Three virus-mediated transfected and 3 episomal vector-mediated transfected iPS cell lines were derived from S-1, S-2 and S-3 somatic cells. Notably, derivation efficiency was lower in S-2 cells for all methods used. When viral transfection was applied, eleven small colonies were observed in 10^6^ S-2 cells, which is a lower efficiency than observed for S-1 and S-3 cells (42 small colonies with 10^6^ S-1 cells and 56 with 10^6^ S-3 cells) (Fig. 7A). When episomal vector transfection was applied, seventeen colonies were found in a total of 10^6^ S-2 cells, whereas sixty-five and seventy-two colonies were obtained in 10^6^ S-2 cells and S-3 cells, respectively (Fig. S2 A). The iPS cell lines established by both methods showed normal morphology and AP activity. Moreover, they showed positive expression of a stem cell surface marker (TRA-1-60) and a pluripotent cell marker (OCT-4). Furthermore, these cells could differentiate into derivatives from all three germ layers both *in vitro* and *in vivo* (Fig. 7B–I).

### Chromosomal analysis for iPS small colonies

**Figure 8:**
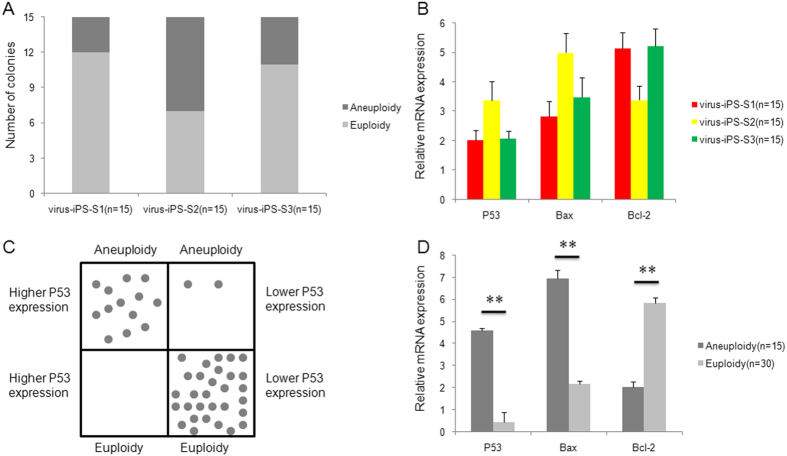
Expression of apoptosis-related genes and chromosome euploidy in arrested primary colonies during virus-mediated iPS induction from different somatic cells. (**A**) Aneuploidy was observed in significantly higher numbers of arrested colonies from virus-mediated iPS-S2 than in colonies from virus-mediated iPS-S1 or virus-mediated iPS-S3; (**B**) Increased expression of P53 and Bax and decreased expression of Bcl-2 were observed in arrested colonies from S2 somatic cells, but these differences were less than two-fold; (**C**) P53 gene expression levels were positively correlated with chromosome aneuploidy in arrested colonies during virus-mediated iPS induction; (**D**) Significant increases in P53 and Bax and significant decreases in Bcl-2 gene expression were observed in arrested primary colonies with aneuploid chromosomes compared with those with euploid chromosomes; ** *P* < 0.01.

Fifteen arrested cell colonies were picked randomly from each virus-mediated transfected group, and the individual colonies were divided into two groups. SNP tests performed on one group revealed aberrant chromosomes in three of the arrested colonies in the virus-iPS-S1 group, comparable with the results obtained from virus-iPS-S3 (four arrested colonies), whereas eight arrested colonies displayed chromosomal deletion/duplication in the virus-iPS-S2 group, regardless of chromosome 1 microdeletion (Fig. 8A). Gene expression data revealed higher P53 and Bax and lower Bcl-2 gene expression in S-2-arrested colonies than in S-1 and S-3 colonies, although this difference was less than two-fold among the three groups (Fig. 8B). Furthermore, P53 gene expression profiling data were significantly associated with chromosome aneuploidy or euploidy (Fig. 8C). Assessment of dynamic gene expression in the aneuploid and euploid colonies revealed at least a three-fold difference between the two groups (Fig. 8D).

We also picked 15 arrested cell colonies randomly in each episomal vector-transfected group and tested their chromosome configuration and gene expression using the method described above. The results were similar to those of the virus-mediated transfection group. Two arrested colonies displayed chromosomal defects in both episomal-iPS-S1 and episomal-iPS-S3 groups, whereas corresponding chromosomal deletion/duplication was shown in 6 arrested colonies of episomal-iPS-S2 (Fig. S2 B). The average expression level of apoptotic genes in S-2 arrested colonies was still significantly different from the other groups (Fig. S2 C). Moreover, P53 gene expression was positively associated with chromosome ploidy (Fig S2 D). Expression of apoptotic genes in aneuploid arrested colonies here displayed much more significant differences from the euploid arrested colonies (Fig. S2 E).

### Chromosome analysis for ES and iPS cells

All ES and iPS cells were propagable until passages 33–40. At passages 10 and 30, five colonies were chosen randomly from the hES-F1, hES-F2, virus-iPS-S1, virus-iPS-S2 and virus-iPS-S3 cell lines, respectively. SNP results revealed no chromosome segment mutation in S-1 and S-3 virus-iPS cells and fertilized ES cells at passage 10, whereas one colony in virus-iPS-S2 showed a 25 Mb duplication in chromosome 12, with the chromosome 1 microduplication. At passage 30, no aberrant chromosome configurations in hES-F1, hES-F2 and virus-iPS-S1 were detected. However, microdeletion was observed in one colony of virus-iPS-S3, and three colonies in virus-iPS-S2 showed chromosomal deletion/duplication, regardless of chromosome 1 microduplication (Table S4).

## Discussion

In the present study, we describe chromosomal mutations induced as a result of two commonly used reprogramming methods, specifically, SCNT and forced ectopic expression of defined transcription factors. The results indicate that aneuploidy in cloned embryos is induced by micromanipulation during SCNT as well as in donor cells with chromosome microduplication. A correlation was evident between aneuploidy and apoptosis-related gene expression, but no correlations were observed for embryo morphology. No cloned ES cells were obtained, and all arrested cloned primary colonies displayed aneuploid chromosomes and dynamic expression of apoptosis-related genes. Among the three iPS cell lines derived from different somatic cells, aneuploidy was observed in all of the arrested primary colonies. The aneuploidy rate was increased upon reprogramming of the S-2 cells, resulting in lower efficiency of somatic cell production.

The traditional cytogenetic method has been applied in the clinic for several decades, and G-banded karyotyping is the gold standard for assessing cellular cytogenetic stability based on a maximum resolution of 5–10 Mb^18^. Karyotyping in different passages is considered a key criterion for establishing normal ES or iPS cell lines, and SNP analysis represents a powerful technique for the identification of small genomic abnormalities greater than 400 kb^19^ using single cells. The advantages of SNP analysis has led to its application in pre-implantation genetic diagnosis/screening and prenatal genetic testing. In the present study, some minor duplications/deletions that were less than 5 Mb could not be identified using the G-banding cytogenetic method but were successfully detected using SNPs.

Chromosomes from all donors were identified as normal before SCNT and iPS manipulation. Therefore, chromosomal aberrations in S-2 somatic cells were assumed to originate from *in vitro* isolation and culture because chromosomes appeared normal even at passage 3. Long-term *in vitro* culture has been shown to impair chromosome euploidy in mesenchymal stem and ES cells^20^,^21^,^22^, but this rarely occurs in human somatic cells. Shao *et al.* suggested that suppression of mitotic recombination is the predominant mutational pathway in somatic cells^23^. Moreover, Cervantes and co-workers reported lower mutational frequency in ES than somatic cells^24^. Xue and colleagues observed that human dermal cells show a higher risk of genetic anomalies, such as increased number of X chromosomes, at high passage numbers^25^. Therefore, it is possible that chromosome segment duplication is induced by treatments for isolating and culturing S-2 cells, including enzymatic digestion, which has been shown to convey predisposition to chromosomal mutation in ES cells.

SCNT is one of the most important current procedures for therapeutic cloning. Several laboratories worldwide have reported the successful development of human cloned blastocysts^26^,^27^,^28^. However, Mitalipov *et al.* were the only researchers to successfully establish ES cell lines from these blastocysts^5^. Therefore, to improve cloning techniques, the key factors blocking the transformation of human cloned blastocysts to ES cells should be explored. In the present study, a significant proportion of arrested eight-cell embryos showed varying degrees of chromosomal mutation. Aneuploidy is suggested to play a key role in the blocking of embryo development in assisted reproductive technology^15^. Consistent with this theory, our data confirmed a correlation between aneuploidy and apoptosis. Yu *et al.* (2007) suggested that inappropriate injection methods in SCNT induce micronucleus formation and apoptosis in mouse cloned embryos, resulting in arrest at the two-cell stage^16^, which involves zygotic genome activation and is comparable to human embryos at the eight-cell stage^29^. In a study by Mitalipov and co-workers, virus-mediated fusion, instead of traditional injection or electrofusion methods, was shown to be a key factor for acquiring high-quality human cloned blastocysts^5^.

Here, we did not observe a correlation between aneuploidy and embryo morphology. A high rate of aneuploidy has been observed in poor-quality human fertilized embryos arrested at the eight-cell stage^15^,^30^. Malmgren *et al.* reported that aneuploidy also occurs in good-quality embryos^31^. Occasionally, microdeletion/duplication in chromosomes can be repaired, which allows aneuploid eight-cell embryos to display euploidy when they develop to the blastocyst stage but fails to eliminate paternal chromosomal defects in human fertilized embryos^32^. However, this was not observed in our study. We propose that aneuploidy results from the process of somatic cell reprogramming, which is not a normal developmental step.

During ICM transformation into ES cells, the quality of primary colonies is a key factor. Campbell and colleagues suggested that increasing the number of epiblast cells and primary colonies can improve human ES cell derivation^33^. Wakayama’s group confirmed differentiated expression of OCT-4 and NANOG in primary colonies, which limited the derivation of cloned ES cells, supporting the importance of primary colonies^34^.

iPS cells from mouse somatic cells were produced using virus-mediated transfection method in 2006 by Yamanaka^6^, and they were followed by iPS cells from rat^7^, pig^35^, monkey^8^ and even human^9^. Regarding genomic integrity when virus-mediated transfection method was applied, Yu *et al.* developed a non-viral method using episomal vectors^36^. In our study, two representative methods were applied to determine whether iPS cell derivation or donor somatic cells resulted in the chromosome defects in iPS arrested colonies. iPS cells were derived from S-1, S-2 and S-3 somatic cells, despite the chromosome 1 segment duplication in S-2 cells. Aneuploid, even triploid pluripotent stem cells, have been established in humans, and they display the general characteristics of diploid human ES or iPS cells^37^. S-2 iPS cells also expressed all human stem cell markers, suggesting that chromosome aberrations are independent of iPS cell derivation and their characteristics. However, the decreased number of iPS cell colonies in S-2 somatic cells during the reprogramming process indicates that chromosome aberrations are related to the efficiency of iPS cell derivation. Mbd3 deletion results in almost 100% efficiency of iPS derivation. Mbd3 is a subunit of NuRD that regulates P53/p21 expression via epigenetic modification^38^. McNamee and colleagues showed that P53-independent apoptosis was closely related to DNA damage-induced aneuploidy^39^, which was consistent with our results.

Reprogramming in iPS cells may have induced point mutations into the genome. Half of these mutations already existed in fibroblast progenitors, whereas the rest occurred during or after reprogramming^40^. In our three established iPS cell lines, two contained normal chromosomes, whereas cells from S-2 somatic cells displayed increased aberration frequency in chromosome segments, suggesting that pre-existing mutations in somatic cells increase the risk of genome instability, which was also recently demonstrated by Letourneau *et al.* in a study of Down’s syndrome^41^. To date, no studies on human ES cells from cloned embryos have been documented, but their characteristics in long-term culture require examination before clinical application.

iPS cells or ES cells produced by SCNT have shown tremendous potential in regenerative medicine and drug screening for certain complex diseases. The present study indicates that reprogramming efficiency was positively correlated to the chromosome microdeletion/duplication, and therefore new methods should be developed to produce such pluripotent stem cells. However, this result also suggests that chromosome defects in somatic cells can induce further chromosomal mutations with the passaging of iPS cells. Thus, for patients with chromosome genetic diseases, the iPS cells from earlier passages will be most useful, and the chromosome configuration must be identified before use.

In conclusion, data from the present study provide insights into the reasons underlying the lower efficiency of human cells reprogrammed using SCNT or iPS methods from the viewpoint of chromosome euploidy and the improvement of cell development efficiency. Our data suggest that pre-existing chromosomal mutations in somatic cells decrease the reprogramming efficiency and increase the incidence of additional chromosomal aberrations in iPS cells, which is a major limitation in their potential clinical application.

## Materials and methods

All chemicals were obtained from Sigma Chemical Co. (Sigma, St. Louis, MO, USA) unless otherwise indicated.

### Ethical guidelines

The present study was approved by the Institutional Review Board at the Peking University Third Hospital, Beijing, People’s Republic of China. All ART and therapeutic cloning procedures closely followed the guidelines legislated and posted by the Ministry of Health of the People’s Republic of China, including the ‘Technical Regulation for Human Assisted Reproductive Technology’ and ‘Ethical Guiding Principles for the Research of Human ESCs’. The methods used in the present study were carried out in accordance with the approved guidelines.

All egg donors and somatic cell donors were informed of details of the procedure, including egg utility and research destination. Patients voluntarily signed a series of informed consent documents. All eggs were used in basic scientific research and not for reproductive purposes.

### Oocyte collection

All of the egg donors involved in the present study were accepted for *in vitro* fertilization treatment because of female factors. The egg donors mainly suffered from polycystic ovarian syndrome (PCOS), resulting in the bursting of more basal follicles per natural menstrual cycle and in a controlled ovarian hyperstimulation cycle. After basic physical examination, the women in the IVF procedure were superovulated using gonadotrophin, and the cumulus-oocyte complexes were retrieved when the diameter of the dominant follicle reached 18 mm under transvaginal B ultrasound. After oocyte collection, each woman and her husband were invited to join this study if the number of retrieved oocytes exceeded 25. If they accepted this invitation and were willing to donate 1-5 oocytes, they were given the option to sign an informed consent. The donated oocytes were removed randomly from the total oocytes and were transferred to the basic research laboratory. The surplus oocytes were fertilized using IVF or ICSI methods depending on the indications from the disease clinic. This procedure complied with the ‘Technical Regulation for Human Assisted Reproductive Technology’ of the Ministry of Health of the People’s Republic of China, and it was used in previous published study^27^.

### Somatic cell isolation and culture *in vitro*

The male donors were 18-29 years old and had a family history of type II diabetes. Skin tissues were collected via biopsy after donors voluntarily signed informed consent documents. Skin was placed in cooled phosphate buffer solution (PBS) and cut into sections using sterilized scissors. After digestion using 1 mg/ml collagenase IV for 30 min and rinsing three times, cells were plated onto 3.5 cm culture dishes, cultured *in vitro* and passaged upon reaching 100% confluence. Beginning at passage 3, cells were frozen, stored in liquid nitrogen, and thawed before use. Three somatic cells could be propagated to 15 passages, and these lines were designated as S-1 from donor 1, S-2 from donor 2, and S-3 from donor 3.

### Somatic cell nuclear transfer and embryo culture

Manipulation of human cells using SCNT was performed as described in our previous study^42^. A subset of oocytes was enucleated assisted with a Piezo apparatus (Prime Tech, Japan), and somatic cells were injected indirectly into the ooplasm. Reconstructed oocytes were activated in 5 μM ionomycin, followed by 2 h of 6-dimethyaminopurine (6-DMAP) treatment, and transferred into G1 culture medium (Vitrolife, Sweden). Oocytes with one or two pronuclei were regarded as successfully activated and were examined every 24 h. On day 3, embryos were removed to G2 culture medium (Vitrolife, Sweden) until the blastocyst stage.

Intracytoplasmic sperm injection (ICSI) micromanipulation was performed in the other group of oocytes, and these developed embryos were used as controls.

### iPS cell induction

Two methods were applied to produce iPS cells. For virus transfection method, a commercial kit (SCR544, Millipore, USA) was used and four genes were induced into the somatic cells, including Oct4, Sox2, Klf4 and c-Myc. Approximately 5–8 * 10^4^ fibroblast cells were plated in each well of a 6-well plate, and the cells were incubated overnight in a 37 °C, 5% CO_2_ incubator. On day 1, the number of cells per well of the 6-well plate was counted, and 1 ml fresh culture medium was applied to maintain target cells. After diluting 1 μl of Polybrene transfection reagent into 9 μl of sterile distilled water to create a 1:10 dilution, and adding 5 μl of the diluted reagent into the culture medium, the calculated volume of virus was added to the plate. The viral titre was 3 * 10^8^ IFU/mL, and the desired MOI was 50. On day 2, the transfected cells’ culture medium was changed, and the cells were transfected again using the same conditions. On day 3, the culture medium containing the virus was removed, and 3 ml fresh culture medium was added into each well to maintain the target cells. The culture medium was subsequently changed every two days. On day 6, the transfected cells were digested using 0.25% trypsin, transferred onto prepared inactivated MEF feeder layer, and cultured with human embryonic stem cell culture medium containing DMEM/F12 Media, Knockout^™^ Serum Replacement, Non-essential Amino Acids and β-mercaptoethanol. Thereafter, the culture medium was changed every two days. From day 8 to day 12, small colonies can be observed gradually. On day 12, the transfected cells and old MEF cells were digested and plated onto a freshly prepared MEF layer. On day 18, the compact colonies with defined borders were removed and plated onto the new MEF layer. Human embryonic stem cell culture medium with 10 ng/mL bFGF was added into each well containing primary iPS cells. Colonies were passaged via mechanical cutting every 4–7 days.

For episomal vectors methods, a commercial kit (A14703, Invitrogen, Life Technologies Co., USA) was used. The procedure is generally similar to the viral transfection method, and three episomal vectors were involved in this reprogramming system, which delivered the genes Oct4, Sox2, Nanog, Lin28, L-Myc and Klf4. After the somatic cells reached 70% confluence, the cells were collected into 15 ml conical tubes and treated for electroporation. Next, 3 * 10^6^ cells were transferred into a new 15 ml conical tube containing fibroblast culture medium. After centrifugation, the s supernatant was carefully removed, and the cells were resuspended using Neon^®^ Transfection kits (MPK5000, Neon^®^ Transfection System, Invitrogen, Life Technologies Co., USA) with 3 pulses of 1650 V and 10 ms inter-pulse intervals. After transfection, the cells were plated onto 100 mm vitronectin-coated plates and incubated overnight at 37°C in a humidified CO_2_ incubator. The fibroblast medium was removed on day 1, and 10 ml N2-B27 medium was supplemented with CHALP molecule cocktail and bFGF. Then, the culture medium was changed every two days until day 15 post-transfection. On day 15, the N2-B27 medium was replaced with Essential 8^TM^ medium, and the small colonies could be observed under microscope. Medium changes every other day were resumed. The iPS cell colonies grew within 15 to 21 days after transfection and could be passaged.

### Derivation of ES cells

The inner cell mass (ICM) was isolated via micromanipulation using laser equipment (ZilosTK, Hamilton, USA). Isolated ICM was planted onto prepared feeder cells for approximately 5–9 days, and primary ESC colonies were dissociated mechanically and cultured in human ES medium consisting of 80% Dulbecco’s modified Eagle medium (DMEM) (Gibco, Life Technologies, USA), 20% Serum Replacement (Gibco, Life Technologies, USA), 1 mM glutamine, 1% nonessential amino acid (Gibco, Life Technologies, USA), 0.1 mM 2-mercaptoethanol, 50 UI/ml penicillin and 50 UI/ml streptomycin mixture, as described previously^42^. ES colonies were mechanically dissociated every 4–5 days. All ESCs were cultured at 37 °C, 5% CO_2_ under a humidified atmosphere.

The remaining trophectoderm (TE) cell clusters were pinched in half with a needle. One part was transferred to lysis buffer for SNP testing, and the other was cryopreserved at −80 °C for examination of gene expression.

### SNP testing

Samples were collected from embryos, ES and iPS cells. SNP manipulation was performed according to the manufacturer’s specifications (Illumina, USA). Sample DNA was amplified using the REPLI-g Midi Kit (Qiagen, Germany) and denatured. Denatured DNA samples were placed in a hybridization chamber for 12–24 h and dropped onto chips, which were dried using a vacuum pump. Chips were scanned using a HiScan SQ (Illumina, USA), and the results were analysed using Genome Studio software.

### Chromosome 1 detection using the NGS method

The NGS method was applied to reveal the chromosome 1 defects in S2 cells. The method was based on a previous study^43^. Genomic DNA was extracted from S2 cells, and then the DNA double-strand was broken down. Next, 100 bp DNA fragments were collected to establish the DNA library, and they were end-repaired, dA-tailed, and adaptor ligated before PCR amplification. Sequencing was performed using a HiSeq 2500 machine, and the data were analysed using standard software (Annuoyouda Scientific Company, China).

### Immunofluorescence

To determine stem cell characteristics, we performed staining for pluripotent and cell surface markers. Cells were fixed in 4% paraformaldehyde (w/v) and permeabilized in PBS containing 0.2% Triton X-100 for 20 min at room temperature. After subsequent washes in PBS, embryos were blocked for 30 min at room temperature. Slides were incubated separately with TRA-1-60 (1:200, ab16288, Abcam, UK) and Oct4 (1:200, ab27985, Abcam, UK) antibodies overnight at 4 °C, followed by fluorescein isothiocyanate-conjugated goat anti-mouse or anti-rabbit secondary antibodies (1:200) for 1 h at 37 °C and re-washing in PBS. Nuclei were stained with 4′,6-diamidino-2-phenylindole (DAPI) at a final concentration of 0.01 mg/ml for 5 min. Immunostained embryos were mounted on glass slides and examined under a confocal laser-scanning microscope (A1-R, NIKON, Japan).

### RNA extraction and reverse-transcript PCR

Expression patterns of apoptosis-related genes, including P53, Bax and Bcl-2, were analysed using RT-PCR. Total RNA was extracted using TRIzol. cDNA was synthesized from ~1 mg total RNA using SuperScript II reverse transcriptase (Invitrogen, Life technologies, USA) and subjected to PCR with primers for NF68KD, α-fetoprotein and albumin (Table S5), with β-actin as the control. Amplified products were size-fractionated using 1% agarose gel electrophoresis and visualized with ethidium bromide staining. Final data were analysed in an image analyser (Bio-Rad, USA).

### Determination of differentiation abilities of stem cells *in vitro* and *in vivo*

To investigate stem cell differentiation ability *in vitro*, ES or iPS cells were cultured in suspension, and embryoid bodies (EBs) formed after 7–14 days. Differentiated EBs were collected, and then RNA was extracted from them. Genes from the three germ layers were identified by RT-PCR: NF68KD (ectoderm), HBZ (mesoderm) and albumin (endoderm).

To examine differentiation *in vivo*, colonies of ES or iPS cells were digested with 1 mg/ml collagenase type IV for 10–30 min and dispensed into 300–400 small human ES cell colony suspensions. Cell masses containing 400–500 cells were injected into inguinal grooves of 6 week-old male severe combined immunodeficiency (SCID) mice as described previously. Teratoma formation at 6–8 weeks was identified using haematoxylin and eosin staining. Light microscopy was used for assessment of the presence of derivatives from the three germ layers.

### Statistical analysis

Results were compared using SPSS 13.0 software (Chicago, IL, USA). The independent samples t-test was used to compare data from two groups, and one-way ANOVA was used for three or more groups. *P* values < 0.05 were considered significant.

## Author Contributions

Y.Y. performed micromanipulation and derived ES and iPS cell lines, and also took part in manuscript drafting, critical discussion and data analysis. L.C. performed SNP and cyto-karytyping experiment, and analyze the data. C.H.Z. performed cell culture and identification, and data collection. R.L. charged for egg or somatic cell donation. Y.F. took part in critical discussion and data analysis, and gave the key advice for the manuscript revision. J.Q. contributed to the conception of design, coordinated the research and manuscript editing.

## Additional Information

**How to cite this article**: Yu, Y. *et al*. Chromosome microduplication in somatic cells decreases the genetic stability of human reprogrammed somatic cells and results in pluripotent stem cells. *Sci. Rep.* **5**, 10114; doi: 10.1038/srep10114 (2015).

## Supplementary Material

Supplementary Information
