# Supplementary material for: Chromosome microduplication in somatic cells decreases the genetic stability of human reprogrammed somatic cells and results in pluripotent stem cells
Source: Sci Rep. 2015 May 12;5:10114. doi: 10.1038/srep10114 (PMC4428033; doi:10.1038/srep10114)
Supplement: Supplementary Information [file srep10114-s1.pdf]

Chromosome microduplication in somatic cells decreases the genetic stability of human reprogrammed somatic cells and resulted pluripotent stem cells

Yang Yu[1,2,3 #], Liang Chang[1 #], Hongcui Zhao[1], Rong Li[1,2,3], Yong Fan[4], Jie Qiao[1,2,3 *]

1 Center of Reproductive Medicine, Department of Obstetrics and Gynecology, Peking University Third Hospital, Beijing, 100191, China

2 Key Laboratory of Assisted Reproduction, Ministry of Education, Beijing, 100191, China

3 Beijing Key Laboratory of Reproductive Endocrinology and Assisted Reproductive Technology, Beijing, 100191, China

4 Key Laboratory for Major Obstetric Diseases of Guangdong Province, the Third Affiliated Hospital of Guangzhou Medical University, Guangzhou, 510150, China

# These authors contributed equally to this work.

* Corresponding authors

Jie Qiao, M.D, Ph.D.

Mailing address: Department of Obstetrics and Gynecology, Peking University Third Hospital, No. 49 HuaYuan North Road, HaiDian District, Beijing 100191, the People's Republic of China

Tel:/Fax: +86-10- 82265080

E-mail: jie.qiao@263.net

Supplementary figure legends

Figure S1 Enlarged image for chromosome dup(1) (q21.1q21.2) of somatic cells from donor 2. The scattered blue points in the left picture indicate the duplication in chromosome 1, and the rectangle in the right picture shows all of the genes in the duplicated region.

Figure S2 Expression of apoptosis-related genes and chromosome euploidy in arrested primary colonies during episomal-mediated iPS induction from different somatic cells. (A) Significantly higher numbers of arrested colonies from episomal-mediated iPS-S2 showed aneuploidy when compared with colonies from episomal-mediated iPS-S1 and episomal-mediated iPS-S3; (B) Increased expression of p53 and Bax and decreased expression of Bcl-2 were observed in arrested colonies from S2 somatic cells, but these differences were less than two-fold; (C) p53 gene expression levels were positively correlated with chromosome aneuploidy in arrested colonies during episomal-mediated iPS induction; (D) Significant increases in p53 and Bax and significant decreases in Bcl-2 gene expression were observed in arrested primary colonies with aneuploid chromosomes compared with those with euploid chromosomes; ** $P < 0.01$.

Table S1 Summary of chromosome karyotyping in blood cells, fibroblast cells at passage 3 and at passage 10 from S1, S2 and S3 donors

Cells	Cytogenetic method	SNP method
S1 blood cells	Normal	Normal
S2 blood cells	Normal	Normal
S3 blood cells	Normal	Normal
S1 fibroblast cells at P3*	Normal	Normal
S2 fibroblast cells at P3	Normal	Normal
S3 fibroblast cells at P3	Normal	Normal
S1 fibroblast cells at P10	Normal	Normal
S2 fibroblast cells at P10	Normal	Chromosomes 1<br>dup(1)(q21.2q21.2)
S3 fibroblast cells at P10	Normal	Normal

*P3 means passage 3, and P10 means passage 10.

Table S2 Summary of 24 genes located at chromosome 1 (q21.1)

No.	Genes	No.	Genes
1	GJA8	2	GPR89B
3	GPR89C	4	PDZK1
5	LOC200030	6	NBPF11
7	FLJ39739	8	PPIAL4B
9	PPIAL4A	10	NBPF14
11	PPIAL4F	12	NBPF15
13	NBPF15	14	NBPF16
15	PPIAL4E	16	NBPF16
17	PPIAL4F	18	LOC645166
19	LOC645166	20	LOC388692
21	FCGR1C	22	HIST2H2BF
23	PPIAL4B	24	LOC728855

Table S3 Chromosome 1 detection using next generation sequencing method

**Sample**	**Result1**	**Result2**	**Size (Kb)**	**Copy Number Variation (CNV)**	**Gain/Loss**
S1-1	46,XY	Normal	Normal	Normal	Normal
S1-2	46,XY	Normal	Normal	Normal	Normal
S2-1	46,XY	Dup(1)(q21.1q21.2)	2510	Seq 1q21.1-1q21.2<br>(142600000-145110000)<br>×3	Duplication
S2-2	46,XY	Dup(1)(q21.1q21.2)	2410	Seq 1q21.1-1q21.2<br>(142520000-144930000)<br>×3	Duplication
S3-1	46,XY	Normal	Normal	Normal	Normal
S3-2	46,XY	Normal	Normal	Normal	Normal

Each sample were repeatedly sequenced twice.

Normal means that no micro-defects were observed in Chromosome 1.

Table S4 Summary of chromosome deletion/duplication in the established pluripotent stem cell lines

Cell lines	Passage 10		Passage 30	
	Aneuploidy	Euploidy	Aneuploidy	Euploidy
hES-F1	0	5	0	5
hES-F2	0	5	0	5
virus-iPS-S1	0	5	0	5
virus-iPS-S2	1	4	3	2
virus-iPS-S3	0	5	1	4

Table S5 Primer sets used for real-time PCR

Gene	Primer Sets(5’ to 3’)
P53	Forward: 5′-GGAAATTTGTATCCCGAGTATCTG-3′<br>Rev: 5′-GTCTTCCAGTGTGATGATGGTAA-3′
Bax	Forward: 5′-AGTAACATGGAGCTGCAGAGG-3′<br>Rev: 5′-ATGGTTCTGATCAGTTCCGG-3′
Bcl2	Forward: 5′-GTGACTTCCGATCAGGAAGG-3′<br>Rev: 5′-CTTCCAGACATTCGGAGACC-3′
NF68KD	Forward: 5′- TCCCCTGAAGAAATTGGTTAAAAT-3′<br>Rev: 5′- GAGTGAAATGGCACGATACCTA-3′
HBZ	Forward: 5′- CTGACCAAGACTGAGAGGAC -3′<br>Rev: 5′- ATGTCGTCGATGCTCTTCAC -3′
albumin	Forward: 5′- TGCTTGAATGTGCTGATGACAGGG-3′<br>Rev: 5′- AAGGCAAGTCAGCAGCCATCTCAT-3′
β-actin	Forward: 5′-GGGAGAAATGGTGGGCG-3′<br>Reverse: 5′-GCCAGTCTGGGATCGTCATC-3′

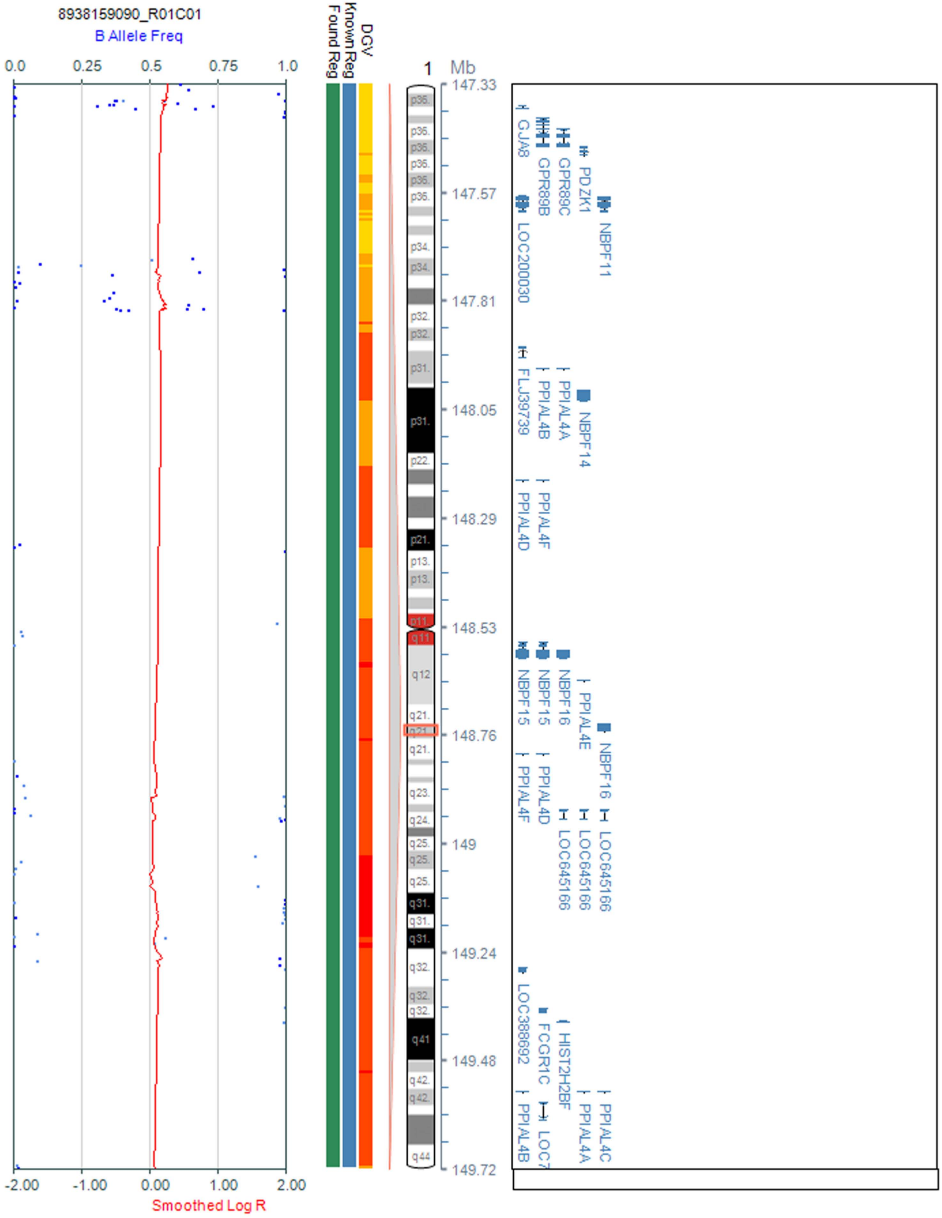

8938159090_R01C01
B Allele Freq
0.0
0.25
0.5
0.75
1.0
-2.00
-1.00
0.00
1.00
2.00
Smoothed Log R
Found Reg
Known Reg
DGV
1
Mb
147.33
147.57
147.81
148.05
148.29
148.53
148.76
149
149.24
149.48
149.72
GJA8
GPR89B
GPR89C
PDZK1
NBPF11
LOC200030
FLJ39739
PPIAL4B
PPIAL4A
NBPF14
PPIAL4D
PPIAL4F
NBPF15
NBPF15
NBPF16
PPIAL4E
NBPF16
PPIAL4F
PPIAL4D
LOC645166
LOC645166
LOC645166
LOC388692
FCGR1C
HIST2H2BF
PPIAL4B
PPIAL4A
PPIAL4C

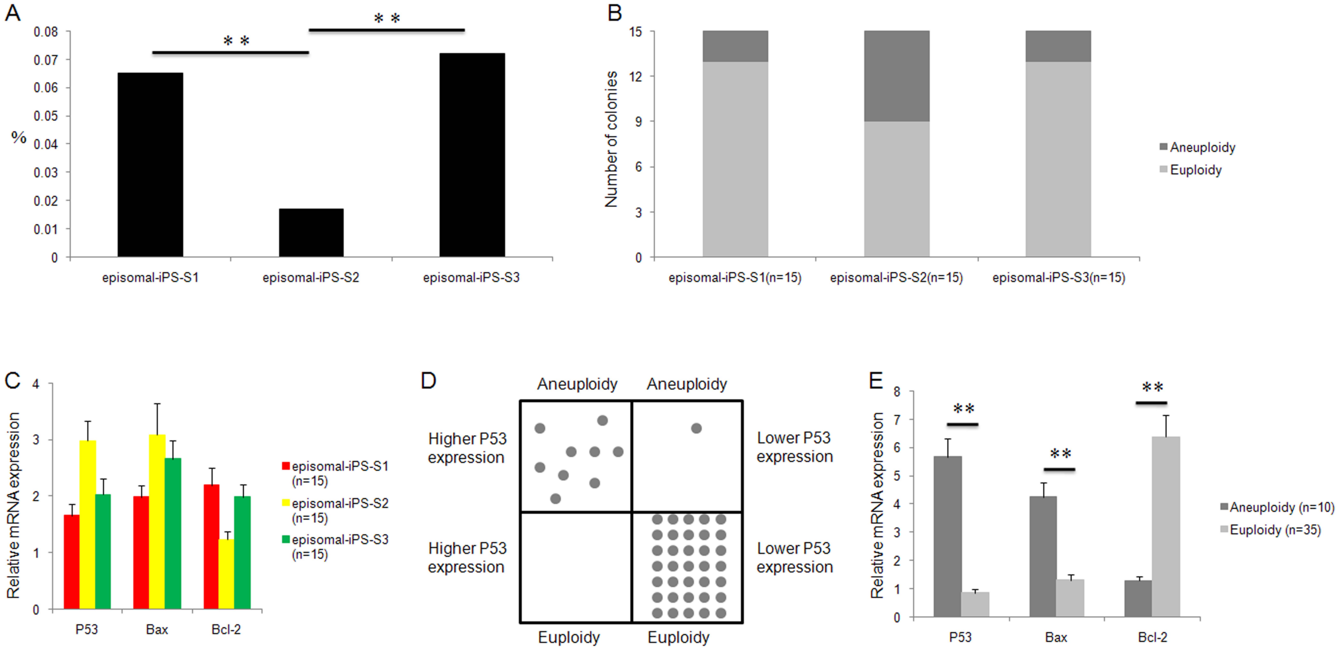

A
%
0.08
0.07
0.06
0.05
0.04
0.03
0.02
0.01
0
**
**
episomal-iPS-S1
episomal-iPS-S2
episomal-iPS-S3
B
Number of colonies
15
12
9
6
3
0
Aneuploidy
Euploidy
episomal-iPS-S1(n=15)
episomal-iPS-S2(n=15)
episomal-iPS-S3(n=15)
C
Relative mRNA expression
4
3
2
1
0
P53
Bax
Bcl-2
episomal-iPS-S1 (n=15)
episomal-iPS-S2 (n=15)
episomal-iPS-S3 (n=15)
D
Aneuploidy
Aneuploidy
Higher P53 expression
Lower P53 expression
Higher P53 expression
Lower P53 expression
Euploidy
Euploidy
E
Relative mRNA expression
8
7
6
5
4
3
2
1
0
**
**
**
P53
Bax
Bcl-2
Aneuploidy (n=10)
Euploidy (n=35)
